# Characterization of an unanticipated indium-sulfur metallocycle complex

**DOI:** 10.1098/rsos.230060

**Published:** 2023-09-20

**Authors:** Joshua J. Morris, Adam Nevin, Joel Cornelio, Timothy L. Easun

**Affiliations:** ^1^ School of Chemistry, Cardiff University, Park Place, Cardiff CF10 3AT, UK; ^2^ School of Chemistry, University of Birmingham, Haworth Building, Edgbaston, Birmingham B15 2TT, UK

**Keywords:** metallocycles, sulfur complexes, catalysis, indium

## Abstract

We have produced a novel indium-based metallocycle complex (**In-MeSH**), which we initially observed as an unanticipated side-product in metal–organic framework (MOF) syntheses. The serendipitously synthesized metallocycle forms via the acid-catalysed decomposition of dimethyl sulfoxide (DMSO) during solvothermal reactions in the presence of indium nitrate, dimethylformamide and nitric acid. A search through the Cambridge Structural Database revealed isostructural zinc, ruthenium and palladium metallocycle complexes formed by other routes. The ruthenium analogue is catalytically active and the **In-MeSH** structure similarly displays accessible open metal sites around the outside of the ring. Furthermore, this study also gives access to the relatively uncommon oxidation state of In(II), the targeted synthesis of which can be challenging. In(II) complexes have been reported as having potentially important applications in areas such as catalytic water splitting.

## 1. Introduction

There has been significant interest in the synthesis of metallocycle compounds due to their potential in sensing [1–3], catalysis [4], molecular magnetism [5,6] and biological applications [7,8]. Metallocycles have been studied in particular as heterogeneous catalysts with very promising results due to the accessibility of active metal sites and unusual oxidation states often found in these relatively large complexes [9–11]. However, many such compounds are made with rare or precious metals and replacing them with more abundant and cheaper alternatives such as indium has become a necessity [12].

Indium compounds such as indium(III) chloride have been used as catalysts for many organic reactions including the hydroarylation of biaryls and cycloisomerization of cyclohexenylalkynes [13]. Indium(III) fluoride and indium(III) triflate promote the cyanation of aldehydes and Diels–Alder reaction of imines [14,15]. Catalysis by indium(II) compounds is relatively uncommon [16]. In(II) selenide has been employed in two-dimensional semiconductors for the fabrication of thin film cells for optoelectronic applications and for water splitting [17,18]. Furthermore, the In(III) state is thermodynamically favoured so the number of reported In(II) compounds is relatively few [19]. Indeed, in this study we were originally using In(III) nitrate as a precursor in reactions intended to make metal–organic frameworks (MOFs), an area of growing interest [20–26], and were surprised to find an In(II) product formed. The synthesis processes of MOFs is an area of particular interest, but relatively little consideration has been given to identifying the side-products when phase-pure materials are not formed [27,28]. After further investigation, we report herein the synthesis and crystal structure of an intriguing and unexpected indium(II)-methanethiolate complex.

## 2. Material and methods

Synthesis of the **In-MeSH** metallocycle is based on a traditional solvothermal route commonly used for MOF syntheses. In our first synthesis of **In-MeSH**, we included an aromatic dicarboxylic acid linker with the intention of forming a MOF. Subsequent reactions showed that the presence of this dicarboxylic acid plays no appreciable role in the formation of the product, since **In-MeSH** forms in the analogous reaction without the dicarboxylic acid present.

Dimethyl sulfoxide (DMSO, 1.0 ml) was added to a solution of indium nitrate hydrate (92.0 mg, 0.198 mmol) in dimethylformamide (DMF, 3.0 ml). To this, 6 M HNO_3_ (100 µl) was added, and the resulting solution was heated in a sealed Wheaton vial at 65°C for 17 h. The reaction yielded pale yellow cubic crystals of **In-MeSH**. The as-synthesized crystals were analysed by powder X-ray diffraction (PXRD) and the crystal structure was determined by single-crystal X-ray diffraction (SCXRD). Attempts to optimize the synthesis showed that the reaction will not proceed unless DMF is present.

Single-crystal X-ray diffraction data for **In-MeSH** was collected on an Agilent SuperNova Dual Atlas diffractometer at 150 K using Mo K*α* radiation (*λ* = 0.71073 Å). Crystals were immersed in Fomblin before being loaded onto a MiTeGen Kapton micromount. Data collection and processing were conducted using CrysAlisPro. Structures were solved via dual-space direct methods using ShelXT and refined by full-matrix least-squares on F2 using ShelXL-2014 within the Olex2 software. All non-hydrogenic atoms were refined anisotropically; hydrogen atoms were assigned using a riding model only. A weighting scheme and absorption corrections were applied to the structure. The PXRD pattern was collected at room temperature on a X'PertPro PANalytical Chiller 59 diffractometer using CuK*α* radiation (*λ* = 1.5406 Å). The sample was loaded onto a zero-background silicon wafer directly from the reaction solution, with excess solvent being wicked away with tissue paper before the measurement.

## 3. Results

**Figure 1.**
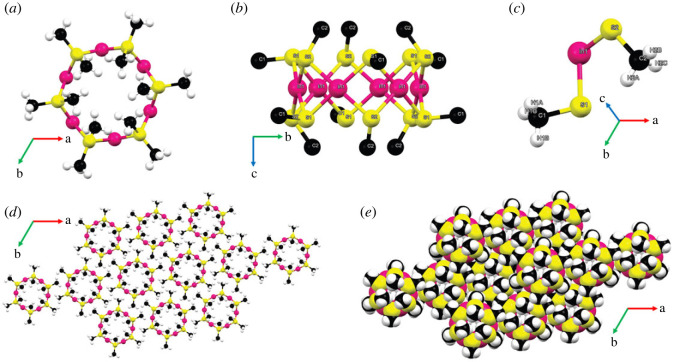
(*a*) The metallocycle viewed along the *c*-axis. (*b*) The metallocycle viewed along the *a*-axis, with the atom labels shown and hydrogen atoms removed for clarity. (*c*) The asymmetric unit. (*d*) Two-dimensional packing viewed along the *c*-axis. (*e*) Space filling diagram of **In-MeSH** showing no voids. Colour code: pink: indium, yellow: sulfur, black: carbon, white: hydrogen.

In table 1, analysis by single-crystal X-ray diffraction shows that **In-MeSH** crystallizes in the rhombohedral *R*-3 space group, with unit cell parameters a = b = 17.6632 (9) Å, c = 8.7095 (5) Å, and a unit cell volume of 2353.2 (3) Å^3^. The metallocycle asymmetric unit comprises two components: an indium ion and two deprotonated methanethiol (CH_3_S^−^) groups which bridge adjacent indium ions to yield an In_6_(CH_3_S)_12_ metallocycle (figure 1).

**Table 1.** Crystal structure data for **In-MeSH**.

*crystal data*	
chemical formula	C_12_H_36_In_6_S_12_
Mr	1254.05
crystal system, space group	Trigonal, *R*-3
temperature (K)	150.01
a, c (Å)	17.6543 (8), 8.7083 (4)
V (Å^3^)	2350.5 (2)
Z	3.0
radiation type, *λ* (Å)	Mo K*α*, 0.71073
*μ* (mm^−1^)	0.90
F (000)	1782.0
Dx (Mg m^−3^)	2.658
*Θ*min., *Θ*max. (°)	3.5, 29.1
*data collection*	
diffractometer	SuperNova-Duo, Atlas diffractometer
absorption correction	Multi-scan CrysAlisPro SCALE3 ABSPACK
Tmin., Tmax.	0.933, 1.0000
measured reflections	4331
independent reflections	1326 [*R*_int_ = 0.0209, *R*_sigma_ = 0.0233]
reflections with I > 2*σ*(I)	1225
*refinement*	
R[F2 > 2*σ*(F2)]	0.0240
wR(F2)	0.1071
S	1.12
data/restraints/parameters	1326/0/48
hydrogen treatment	H-atom parameters constrained
largest diff. peak/hole (e Å^−3^)	0.82/−0.58

Each indium ion is coordinated to four sulfur atoms, one from each of the four deprotonated methanethiol molecules. These sulfur atoms each bridge two indium ions, giving a metallocycle ring that exhibits a distorted square planar coordination around each indium centre; the S1-In-S2 angle is 97.7°, while the S1-In-S1 angle is 81.9°. Furthermore, viewing the metallocycle along the *c*-axis shows the S1-In-S1 and S2-In-S2 angles to be 174.5° and 178.5° out of the plane of the four coordinated sulfur atoms. The In–S distances in **In-MeSH** are between 2.321 and 2.332 Å, which are shorter than both In(III)–S bonds (typically approx. 2.4–2.5 Å) and In(I)–S bonds (typically approx. 2.7 Å) [29–31]. The In–In distance is 3.214 Å, eliminating the possibility of In–In bonds, typically reported to be 2.7–2.8 Å [16].

We compared these bond lengths and angles of **In-MeSH** with its Ru, Zn and Pd analogues, hereby called **Ru-MeSH**, **Zn-MeSH** and **Pd-MeSH**, respectively (electronic supplementary material, tables S1–S3) [32]. All these metallacycles crystallize in the same *R*-3 space group except for **Zn-MeSH**, which belongs to the P2_1_/n space group. **In-MeSH**, **Ru-MeSH** and **Pd-MeSH** show very similar unit cell lengths (a = b ≈ 17.6–17.8 Å and c = 8.7 Å). For all four of these metallacycles, the M–S bond lengths (approx. 2.3 Å) and M–M distances (approx. 3.1 Å) are also relatively similar (electronic supplementary material, figure S1). In reported structures containing M–M bonds, we observe example bond lengths for Ru–Ru, Zn–Zn and Pd–Pd bonds to be 2.848, 2.358 and 2.6–2.7 Å, respectively [33–35]. Comparing these bond lengths with the M–M distances of the respective metallocycles leads us to conclude that the four metallacycles do not possess M–M bonding.

## 4. Discussion

**Figure 2.**
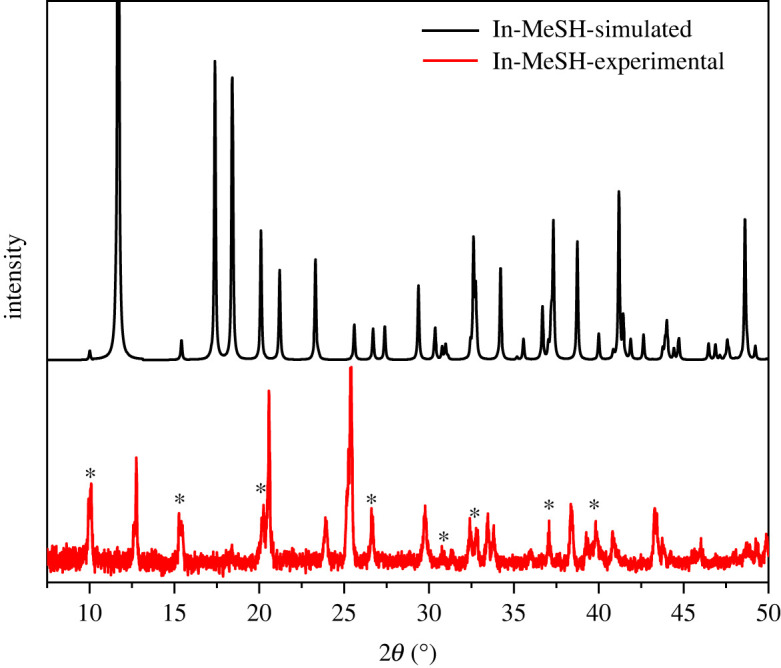
A comparison of the PXRD pattern of synthesized **In-MeSH** (red) to the PXRD simulated from the SCXRD (black). Asterisks indicate peaks that match with the simulated PXRD.

The synthesis forms a solid product, within which crystals of sufficient quality to obtain the single crystal structure were identified. However, we also simulated a PXRD pattern from the SCXRD structure and compared it with the experimental pattern of the bulk powder material (figure 2) to find that **In-MeSH** does not form as a phase-pure solid. Peaks at 2*θ* = 10.1°, 15.2°, 18.3°, 20.2°, 26.6° and 39.8° match those reflections simulated from the SCXRD structure, but comparison of the additional observed peaks against indium nitrate hydrate, indium sulfide, cubic and rhombohedral indium oxides, and indium oxyhydroxide did not afford any matches [36]. We suspect that the relatively uncommon In(I) or In(II) oxides may be formed as the other phase in this reaction [37–39] but could not source crystal structures or powder diffraction patterns for either. Furthermore, while the precursor indium nitrate hydrate no longer appears to be present, there may be solid unreacted indium nitrate present as a different solvate than at the start of the reaction.

The analogous Ru complex has an absorption band at 365 nm ascribed to an ligand-to-metal charge transfer (LMCT) transition [32]. This LMCT band tails off in the blue region, around 420 nm, which gives the Ru-complex a yellow colour. For **In-MeSH**, while the metal-based orbitals will certainly be different, if the lowest unoccupied molecular orbital (LUMO) is primarily metal-based across the indium ions, a charge transfer transition may again be the source of the pale-yellow colour observed for these crystals [40]. A full spectroscopic characterization was unfortunately not possible with the limited amount of material available but efforts are underway to make phase-pure **In-MeSH** and perform both experimental and computational characterization of its optical and electronic properties.

**Figure 3.**
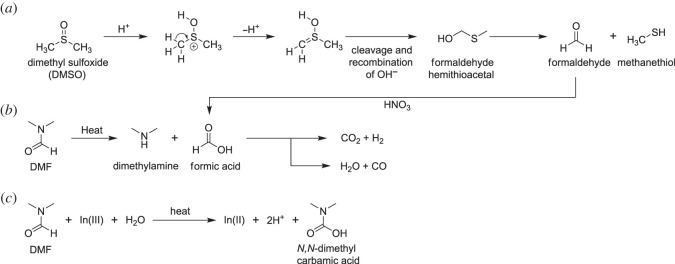
(*a*) Acid catalysed decomposition of DMSO generates methanethiol *in situ*, which subsequently binds to In(II) ions to form **In-MeSH**; (*b*) DMF thermally decomposes, forming hydrogen or carbon monoxide which in turn could reduce In(III) to In(II); (*c*) DMF oxidation to *N,N*-dimethyl carbamic acid could simultaneously reduce In(III) to In(II).

As **In-MeSH** contains methanethiol, which was not one of the initial reactants, the solvothermal conditions used must generate methanethiol *in situ*. If this is a slow or low-yielding process, that may explain the observed low yield and phase-impure solid product. The decomposition of DMSO into methanethiol (figure 3*a*) via an acid-catalysed Pummerer rearrangement has been well studied [30,41–43]. In the presence of strong acids such as HNO_3_, DMSO forms formaldehyde hemithioacetal, which decomposes to methanethiol and formaldehyde. We propose that this is the source of the methanethiol which reacts with In(II) ions, the origin of which are discussed below.

The 2+ oxidation state of the indium ions was determined by inspection of the asymmetric unit, which consists of one indium ion and two methanethiol molecules, each of which formally contributes a 1− charge. It is notable that the reaction only proceeds in the presence of DMF and hence we suspect that DMF is responsible for the reduction of indium (III) to indium (II). Though we could not find specific literature for the DMF reduction of In(III) to In(II), the utility of DMF for the reduction of metal ions is well known, with reported examples including the reduction of Au(III) to Au(0), Ag(I) to Ag(0), W(VI) to W(IV), Pd(II) to Pd(0) and, most pertinently given the existence of an analogous isostructural ruthenium metallocycle, Ru(III) to Ru(II) [44–51]. As suggested by Nagata & Obora, under heating DMF could react via two methods: (i) thermal breakdown via formic acid into either water and carbon monoxide (figure 3*b*) or carbon dioxide and hydrogen, the latter of which in both cases serves as the reducing species, or (ii) DMF reduces the In(III) to In(II), being oxidized to *N,N*-dimethyl carbamic acid in the process (figure 3*c*) [45]. Our reaction medium is strongly acidic, in principle inhibiting the second of these options. As a third alternative, formaldehyde generated from decomposition of DMSO (figure 3*a*) could also be oxidized by HNO_3_ to formic acid, potentially providing another route to reducing In(III) to In(II), but the absence of **In-MeSH** formation when DMF is not present makes this appear a less likely pathway.

The metallocycle itself, it is worth noting, has no pores or voids capable of storing any residual molecules of the solvents DMF or DMSO. Packing in the crystal structure appears to be via weak van der Waals interactions and no stronger supramolecular interactions are apparent. We have compared the crystal structure of **In-MeSH** with its zinc, ruthenium and palladium analogues [32]. As for **In-MeSH**, the Zn(II), Ru(II) and Pd(II) analogues show distortions from the ideal square planar angle of 90° to about 97.7° around each metal centre. The M(II)-S bond length in all four metallocycles is between 2.2 and 2.3 Å. An astute reviewer of this manuscript suggested that perhaps we had made the **Pd-MeSH** analogue as a result of trace Pd-impurities carried through from linker synthesis via cross-coupling reactions. The existence of **Pd-MeSH** forming as a side product in MOF reactions when Pd-catalysed cross-coupling reactions are used for ligand synthesis certainly cannot be ruled out in such cases. The unit cell lengths of the Pd(II) analogue, **Pd-MeSH** are indeed very similar to **In-MeSH** (electronic supplementary material, table S1). However, small differences exist between the S2-M-S2 angles (electronic supplementary material, table S3) and between the M–M distances: 3.214 Å for **In-MeSH** and 3.126 Å in **Pd-MeSH** (electronic supplementary material, figure S1). Since we are able to synthesize **In-MeSH** without any organic ligand present we rule out this possibility.

The Ru analogue is reported to be catalytically active for the alkenylation of aryl pyridines [32]. Though there are not many reports of catalysis by In(II) compounds, we believe that due to the comparatively high abundance of indium in the Earth's crust and the significantly lower cost compared with ruthenium (*ca* 250 times more abundant and *ca* 100 times cheaper) [52,53], **In-MeSH** is a good candidate for future catalyst testing. The catalytic activity of the analogous ruthenium metallacycle is ascribed to the vacant axial coordination site, present again here in the indium-based structure. If obtainable as a phase-pure material, **In-MeSH** may also serve as an effective reducing agent due to the fact that the In(III) state is thermodynamically preferred [19].

## 5. Conclusion

An unanticipated indium(II)-based metallocycle compound has been synthesized using indium nitrate, DMF, HNO_3_ and DMSO. This compound may well be a common side product in many MOF reactions, as it is formed via the partial decomposition of the DMSO solvent in an acidic environment. The unusual metalloband structure contains indium ions in the relatively uncommon 2+ oxidation state and their distorted square-planar geometry means they are potentially accessible by solvents or catalytic substrates in an analogous manner to the previously reported ruthenium structural analogue. Experiments to synthesize phase-pure **In-MeSH** and investigate catalytic activity are underway.

## Data Availability

The crystallographic dataset supporting this article has been uploaded in the electronic supplementary material and has been submitted to the Cambridge Crystallographic Data Centre, reference CCDC 2235845. These data can be obtained free of charge via https://www.ccdc.cam.ac.uk/structures/. The data are provided in electronic supplementary material [54].
